# A Simple Survey of the Preparation Situation for Resident's Evacuation in Japanese Prefectures After the Fukushima Daiichi Nuclear Power Plant Accident

**DOI:** 10.3389/fpubh.2020.496716

**Published:** 2020-10-02

**Authors:** Takakiyo Tsujiguchi, Mizuki Sakamoto, Tomoki Koiwa, Yoko Suzuki, Kouya Ogura, Katsuhiro Ito, Kanako Yamanouchi, Ikuo Kashiwakura

**Affiliations:** ^1^Graduate School of Health Sciences, Hirosaki University, Hirosaki, Japan; ^2^Center for Radiation Support and Safety, Hirosaki University, Hirosaki, Japan; ^3^Advance Emergency and Critical Care Center, Hirosaki University Hospital, Hirosaki, Japan

**Keywords:** nuclear disaster, Fukushima Daiichi nuclear power plant accident, nuclear disaster response system, resident evacuation, human resource development

## Abstract

The Japanese government formulated the Nuclear Emergency Response Guidelines in response to the Fukushima Daiichi Nuclear Power Plant accident (FDNPP accident) caused by the Great East Japan Earthquake in March 2011. Under these guidelines, Japan has established its current nuclear disaster response system. This manuscript outlines the transition of Japan's nuclear disaster response system before and after the FDNPP accident and also shows the results of a questionnaire survey on the level of preparation the prefecture currently has for the evacuation of residents at the time of a nuclear disaster. About 70% of the prefectures where nuclear facilities are located or adjacent have completed or are in the process of completing evacuation plans, and all except one indicated they have the equipment needed to perform radiation contamination inspections of residents. These results suggest that activities are taking place throughout Japan to build a new disaster response system. It will be important to verify whether the evacuation manuals prepared by prefectural governments are effective through large-scale training and to develop human resources for performing radiation contamination inspections of evacuating residents.

## Introduction

In March 2011, the Great East Japan Earthquake caused a Fukushima Daiichi Nuclear Power Plant (FDNPP) accident (1–3). There were no acute radiation syndrome (ARS) victims of the FDNPP accident, but it was classified as an International Nuclear Event Scale (INES) level 7, which mandated a large-scale residential evacuation because of the radioactive materials released into the atmosphere. The Japanese governmental report indicated that about 170,000 residents were evacuated as of May 2011, which had various adverse effects on people, particularly on elders and hospitalized patients (1, 4). About 2,000 people were identified as victims of disaster-related deaths caused by the evacuation's influences that worsened their underlying illnesses (1). Some residents who were afraid of radiation risks left their families and communities, resulting in lifestyle and mental health changes (1, 5–7).

The FDNPP accident was a complex disaster resulting from an earthquake, a tsunami, and it greatly influenced Japan's nuclear disaster response system. The Nuclear Regulatory Authority Japan (NRA) published the Nuclear Emergency Response Guidelines (NERG) in 2012, which included items for which prefectural governments and medical facilities with nearby nuclear plants needed to prepare (8, 9). The 1999 JCO accident (INES level 4) resulted in three ARS patients and the development of laws and establishment of a radiation emergency medical system (10–12); however, the FDNPP accident revealed that those laws and medical systems were unable to respond to complex disasters of its scale. The NERG were designed to ensure that prefectural governments and medical facilities could respond to future complex disasters. It specifically outlined methods for inspecting radioactive contaminations associated with large-scale residential evacuations and networks that allowed regional core hospitals to accept contaminated or exposed patients and evacuated residents (9, 13, 14). In Japan, the nuclear emergency response system began to be established in response to the JCO accident, and the current system was transformed after the FDNPP accident. The nuclear emergency response system in Japan before and after the FDNPP accident will be outlined in detail in section The Transition of Japan's Nuclear Disaster Response System.

**Figure 1:**
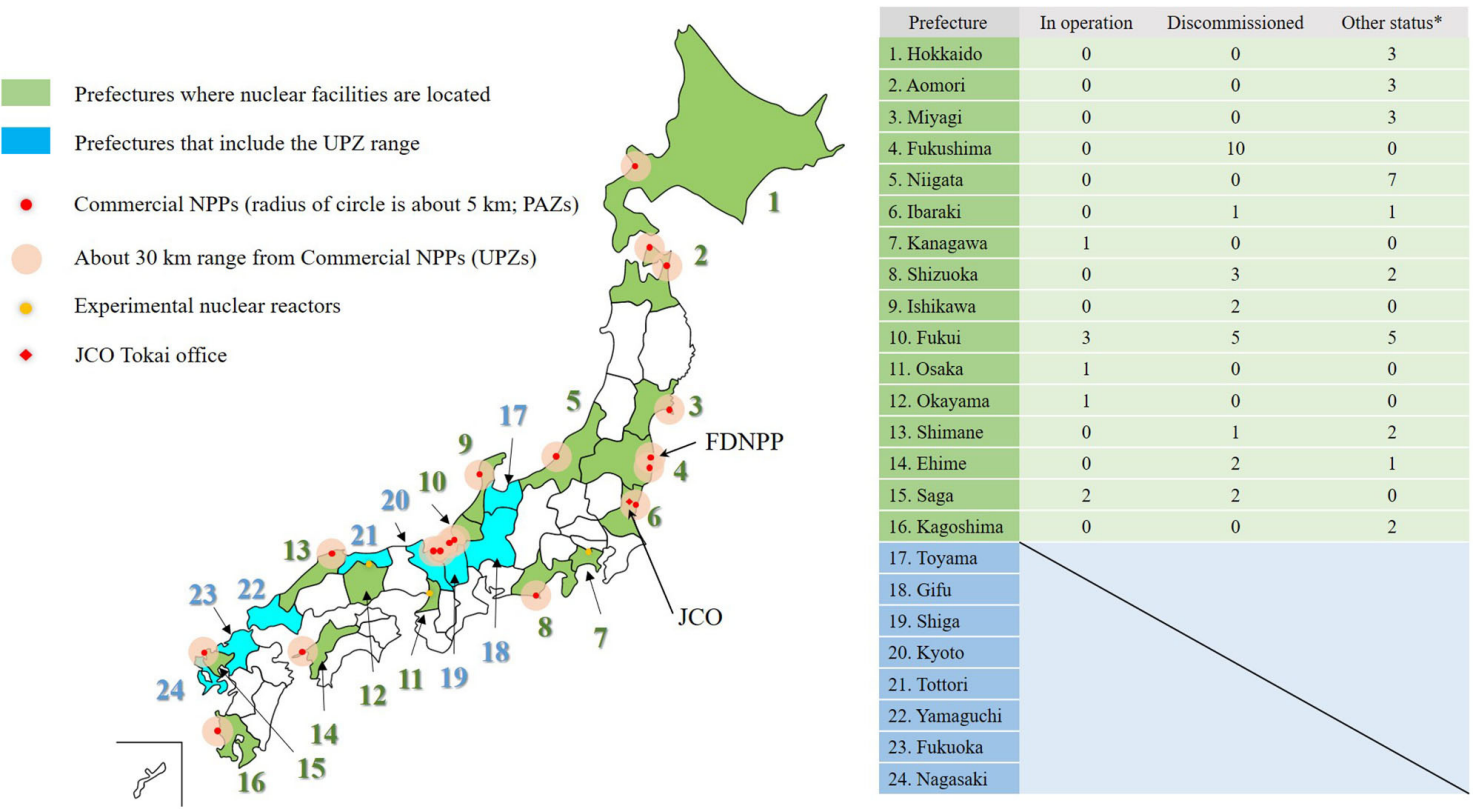
Map of Japan's nuclear facilities and prefectures: Green prefectures have nuclear facilities, blue prefectures do not have nuclear facilities, but they have areas that are within 30 km of an adjacent prefecture's NPPs. It also shows the operating status and number of NPPs as of June 2020. The figure also shows the location of the JCO and the FDNPP. Since JCO is not an NPP, it is not counted in the list on the right. *Other status means preparing for restart or being under inspection.

To understand Japan's nuclear disaster response system, it is important to know where the country's nuclear facilities are located and how many prefectural governments are involved in nuclear disaster response activities. As of June 2020, there were 63 commercial nuclear power plants (NPPs) and experimental nuclear reactors in Japan. Currently, there are eight in operation, 26 with decommissioning decisions, and 29 held for preparation for operation or under review of NRA Japan (15). Of the 47 prefectures in Japan, 16 had nuclear facilities, and eight of the prefectures adjacent to the 16 prefectures with nuclear facilities had Urgent Protective Action Planning Zones (UPZ) about 30 km from the nuclear facilities. UPZ refers to the range of preparations for implementing protective measures, such as indoor evacuation from the stage before radioactive materials are released, in the event of an emergency at NPPs. Thus, 24 prefectures had nuclear disaster countermeasure priority areas that must prepare for medical and evacuation activities in the event of a nuclear disaster (13). Figure 1 shows the Japanese prefectures with nuclear facilities and UPZs.

Nine years have passed since the FDNPP accident, and continued progress vitally depends on the relevant parties' sharing of information about the status and issues of Japan's nuclear disaster prevention system. This paper describes the changes made to Japan's nuclear disaster response system after the FDNPP accident, focused on the prefectural governments' systems and the medical response systems, and it presents the results of an investigation of the extent to which those governments have prepared manuals and equipment regarding contamination inspections. Specifically, section **The Transition of Japan's Nuclear Disaster Response System** of this brief report will introduce the transition of Japan's nuclear disaster response system before and after the FDNPP accident. Then, in section **Survey on the development status of residents' evacuation system after FDNPP accident in Japanese local governments**, we will introduce the results of a questionnaire survey targeting Japanese local governments on the status of preparation of radiation measurement instruments for contamination inspections and the preparation of residents' evacuation plans in the event of a nuclear disaster.

## The Transition of Japan's Nuclear Disaster Response System

### The Nuclear Disaster Response System After the Tokai-Village JCO Criticality Accident (1999-)

Development of the nuclear disaster response system in Japan began with the JCO accident that occurred in 1999 (16). The JCO accident was a radiation accident that was positioned at INES level 4, and three workers were exposed to neutrons and γ rays at close range; two of them died (11, 17). Laws on natural disasters and nuclear reactors have existed since the 1960s, but it was after 1999 that laws on notification of radiation emergencies and medical systems were implemented. The JCO accident triggered the Act on Special Measures Concerning Nuclear Emergency Preparedness, which was enacted in December of that year, as well as ongoing countermeasure efforts in response to radiation emergencies (16). The medical response system in the event of a nuclear disaster comprised a main trunk with branches in which large and mid-sized regional facilities were designated as primary or secondary radiation emergency medical care facilities, and the government was to designate tertiary facilities during an event (12). In Japan, the establishment of the Act on Special Measures Concerning Nuclear Emergency Preparedness set the Emergency Planning Zone (EPZ), the area around a nuclear facility, where protective measures should be implemented, from 8 to 10 km (18). In addition, commercial NPPs are under the jurisdiction of the Ministry of Economy, Trade and Industry, and research reactors are under the jurisdiction of the Ministry of Education, Culture, Sports, Science and Technology. Radiation emergency-oriented disaster response drills were conducted once a year in cooperation with these administrations and local governments, police, fire prevention and other disaster prevention organizations (19). In the nuclear emergency response system before FDNPP accidents, definitions had been established regarding medical treatment and transportation orders when several seriously exposed patients occurred, but large-scale evacuation of residents was not expected.

### The Nuclear Disaster Response System After the FDNPP Accident (2011-)

Since 1999, Japan's nuclear disaster prevention system has gradually been advanced, and it centers on medical institutions and nuclear power plants. Meanwhile, Japan experienced the FDNPP accident in 2011. The FDNPP accident was an accident at a nuclear power plant due to an earthquake and tsunami, and the catastrophic damage to local infrastructure forced evacuations even at the primary radiation emergency medical facilities near the nuclear power plant (1). NRA Japan made the NERG in 2012 in response to this large-scale complex disaster, and the nuclear disaster prevention system has undergone major changes since then. Currently, Nuclear Emergency Core Hospitals have the central role in providing this care to affected areas, are designated as related to particular prefectures, and the medical system has less of a hierarchy than before (9, 12, 20). In addition, the Nuclear Emergency Medical Cooperative Institution, Advanced Radiation Emergency Medical Support Center, and Nuclear Emergency Medical Support Center are now nationally implemented to support human resource development and network construction (9, 14, 21). The Nuclear Emergency Core Hospitals are required to dispatch Nuclear Emergency Medical Assistance Teams in the event of a disaster, whose activities are defined by The Activity Guidelines of Nuclear Emergency Medical Assistance Teams developed and issued by the NRA Japan in March 2017 (9).

**Figure 2:**
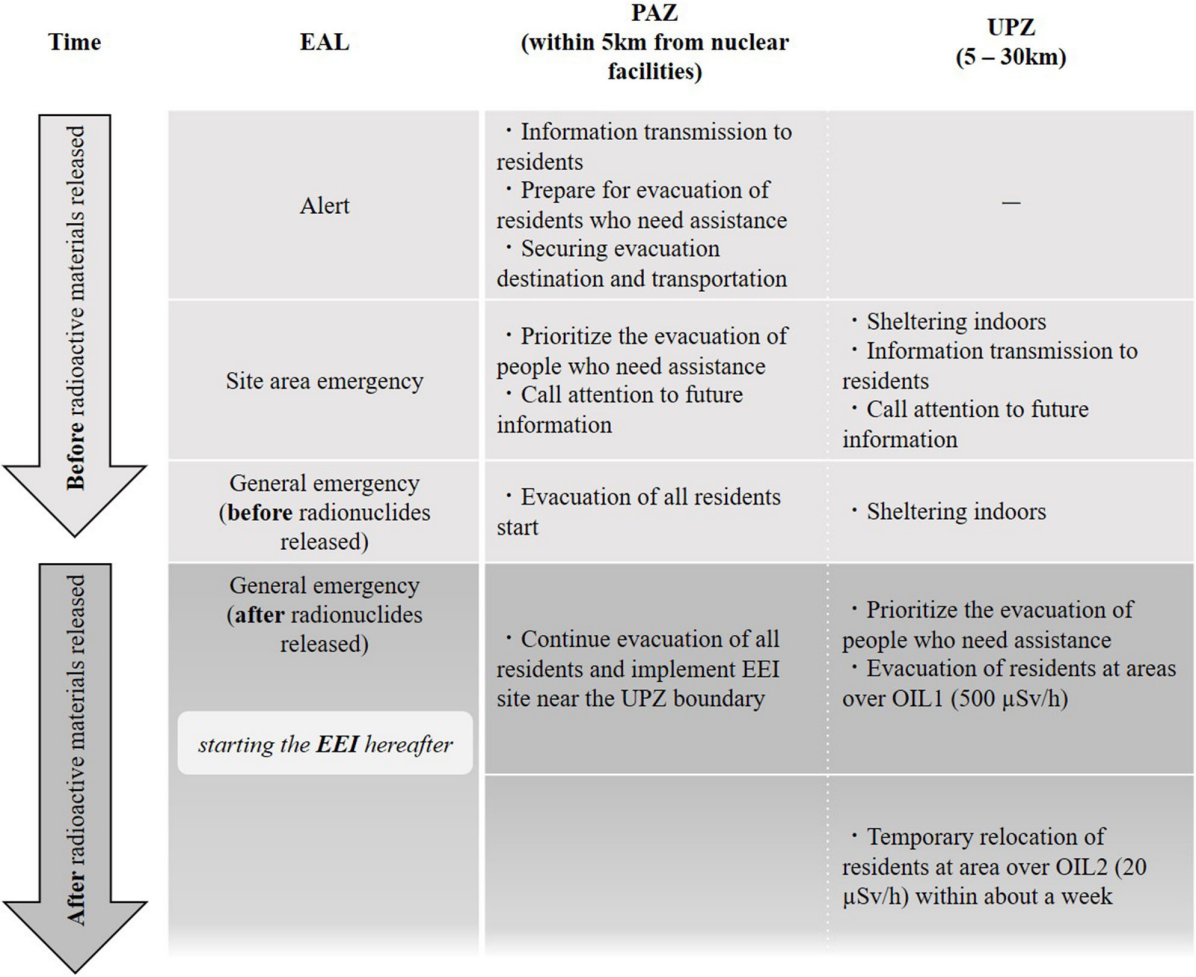
Overview of actions that Japanese local governments should take in response to the EAL indicated in the NERG.

In addition to radiation emergency medicine, NERG also refers to personal and environmental monitoring in the surrounding area. Before the FDNPP accident, the Japanese government had set the EPZ within 8 km to 10 km, but NERG is expanding the EPZ to an area of up to 30 km. Specifically, in the event of an emergency at a nuclear facility, an area where preventive evacuation is started before the release of radioactive materials is called Precautionary Action Zone (PAZ), and an area where protective measures, such as indoor evacuation, are implemented is called UPZ. Figure 2 shows actions that residents in the EPZ should take according to the emergency classification of the nuclear facility and the criteria for environmental and personal monitoring.

To understand Figure 2, it is necessary to know various classifications for emergency situations at nuclear facilities. NERG states that an emergency situation in the event of an anomaly, mainly in a commercial nuclear power plant, consists of three stages: (1) alert, (3) site area emergency, and (4) general emergency (13). These categories are called Emergency Action Levels (EALs); they have a history of being developed according to safety guides indicated by the International Atomic Energy Agency (IAEA) (2). When each stage is announced, residents of the PAZ and UPZ must conduct an indoor evacuation and a wide area evacuation. In addition, in principle, it is mandatory for residents who evacuate the UPZ after radioactive materials are released from NPP to carry out “inspection to confirm whether radiation protection measures should be implemented for evacuees in case of nuclear disaster (Evacuation Exit Inspection: EEI)” (13). In addition, a standard value called Operational Intervention Level (OIL) is applied to personal monitoring mechanisms such as EEI and environmental monitoring. In Japan, NERG shows the standard values for indoor evacuation and various standard values for determining the implementation of residents' decontamination, while referring to the default values set by the IAEA (22). Please refer to the author's past papers for information about specific standard values for OIL in Japan (13).

Japanese local governments which own NPPs and UPZ are starting to prepare evacuation manuals tailored to local conditions such as population and roads so that residents can be systematically evacuated and de-contaminated.

## Survey on the Development Status of Residents' Evacuation System After FDNPP Accident in Japanese Local Governments

### Questionnaire Survey of Prefectures' Preparations for Nuclear Disaster Response

A survey was conducted to assess the progress being made regarding residential evacuation plans mandated for every prefecture as part of Japan's current nuclear disaster response system. The main goal was to verify whether a plan had been established that would allow residents to easily evacuate in the event of a complex disaster similar to the FDNPP accident and whether the prefectures had radiation measurement instruments to use to perform contamination inspections. Questionnaires were mailed in June and July of 2019 to the population of prefectures (*N* = 24) with nuclear disaster countermeasure priority areas. The questionnaire was sent to each local government and collected via e-mail. There were two questions: (1) “Does the prefecture have a regional evacuation plan for residents at the time of a nuclear disaster?” and (2) “Does the prefecture have sufficient radiation measuring instruments (such as GM survey meter, NaI scintillation survey, gate monitor, and so on) using for personal monitoring at the time of EEI?” Approval of the study was obtained from the ethics committee of the Graduate School of Health Sciences, Hirosaki University. The data were tabulated and the extent to which the prefectures were prepared was assessed using a simple frequency analysis.

### Results of the Questionnaire Survey

**Figure 3:**
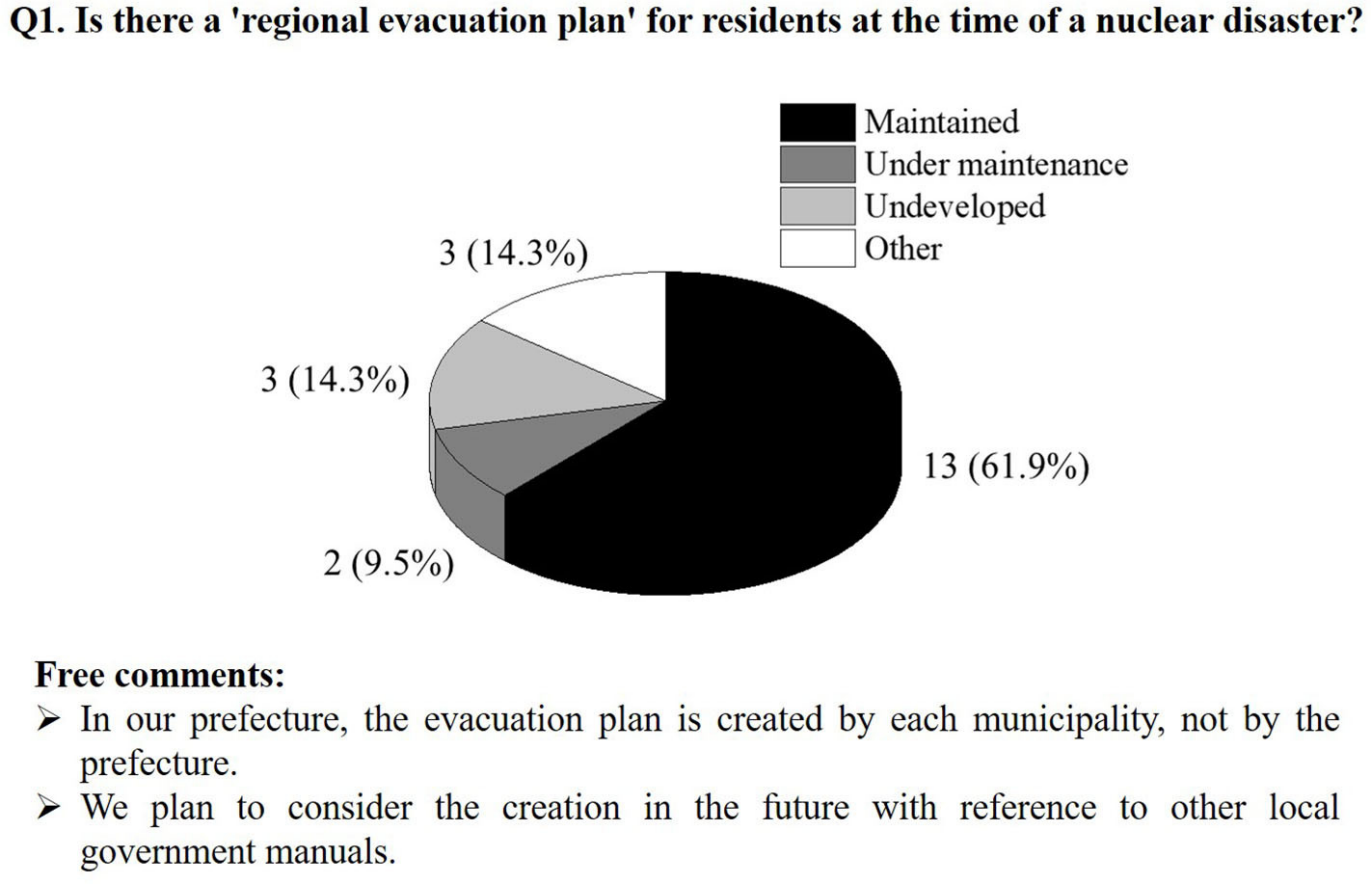
Evacuation plan development status.

**Figure 4:**
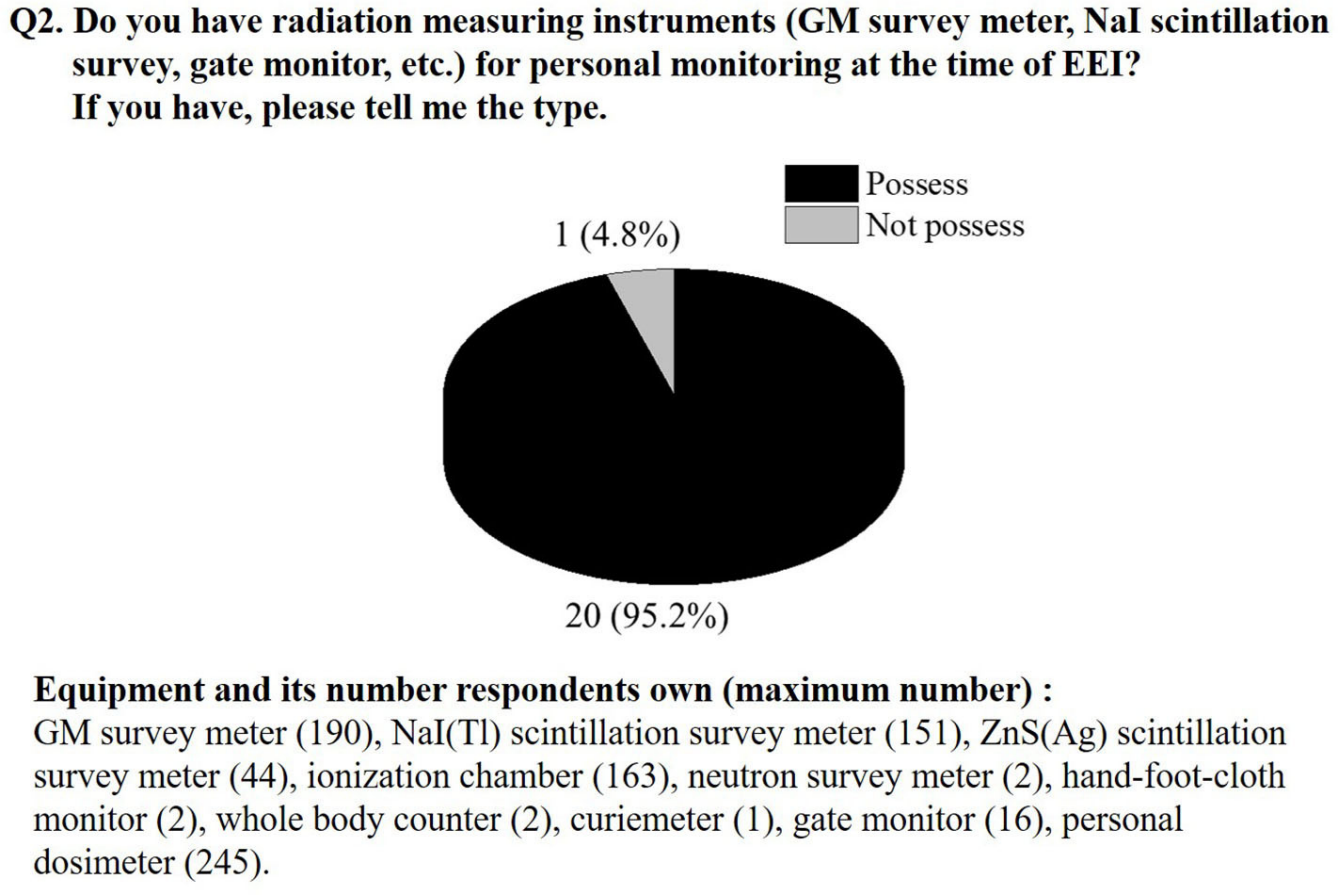
Status of radiation measurement equipment for performing contamination inspections of residents. At the bottom of the figure are the specific names of radiation measurement equipment owned by each local government. The number on the right side of the name indicates the maximum number of holdings among the local governments who answered that they own it.

Participation was voluntary, and 21 of the 24 prefectures provided data by returning completed questionnaires (87.5% response rate). Figures 3, 4 illustrate the results of the questionnaire. Regarding an evacuation plan, about 70% of the prefectures had completed or were developing a plan (Figure 3). The unprepared prefectures reported that they were considering plans for the future and that their municipalities had independently created evacuation plans. Among the local governments that responded, there were some that planned to create preparations in the future with reference to the manuals of other local governments, but most local governments were willing to develop a systematic manual on the evacuation of residents during a nuclear disaster. Figure 4 indicates that just one prefecture did not have sufficient radiation measurement equipment to perform radioactive contamination inspections on residents. In other words, other 20 local governments are preparing the radiation measurement equipment for personal monitoring. In addition, Figure 4 also shows the types and number of radiation measurement devices owned by each local government. Due to ethical issues related to the questionnaire survey, it is not possible to reveal the specific name of the relevant local governments, but most local governments have GM survey meters used for contamination inspection and NaI(Tl) scintillation survey meters for measuring air dose rates.

## Discussion

Nine years have passed since the complex FDNPP accident, and it is important to know the extent to which the Japanese central and prefectural governments have strengthened their nuclear disaster prevention systems. The biggest problems during the FDNPP accident concerned evacuating residents, inspecting contamination, and the evacuees' long-term mental health (23, 24). There are also reports that radioactive contamination from the FDNPP accident caused reputational damage, affecting the industries of foods and products from Fukushima prefecture as well as residents from contaminated areas (25–28). Moreover, when residents near the FDNPP evacuated after the accident, some evacuees were refused entrance to the evacuation shelter because they had no certificates showing they were “radiation free.” It was obvious that a new disaster response plan was needed.

As discussed in section **The Transition of Japan's Nuclear Disaster Response System**, before and after the FDNPP accident, the radiation emergency medical system and resident evacuation system for radiation emergencies in Japan changed significantly. In particular, regarding the resident evacuation system, the current system instituted after the FDNPP accident, which is mentioned in NERG, enables the evacuation and individual monitoring of a wider range of residents. We conducted a simple questionnaire survey on the status of the preparation of manuals for resident evacuation in Japan's local governments and the status of possession of radiation measurement equipment corresponding to the implementation of EEI. Results of the questionnaire survey show that more than 70% of local governments have prepared or are preparing resident evacuation manuals, and the other 30% of local governments are considering undertaking such preparations in the future by referring to the manuals created by other local governments. For example, Hokkaido prefecture, the northernmost municipality of Japan, has released a manual on the Internet regarding the evacuation of residents and information transmission to residents including tourists when a nuclear disaster occurs (29). Hokkaido prefecture has shown concrete actions for residents in PAZ and UPZ corresponding to EAL, and it also specifies the contents of emergency broadcast systems and corresponding actions so that tourists can smoothly evacuate. This resident evacuation manual is a high quality precedent and is of interest to the concerned parties as well as other Japanese local governments. Moreover, it was shown that more than 90% of the local governments possess the radiation measuring equipment necessary for inspecting pollution and measuring the air dose rate. Depending on the region, some local governments have a population of several hundred in the PAZ, and some local governments have a population of several hundred thousand in the UPZ, so there are differences in the types and numbers of radiation measurement equipment they own. The feasibility of EEI in a radiation emergency in each region should now be fully verified through extensive training.

One important future task is to verify the effectiveness of the evacuation manuals regarding radiation contamination inspections of residents prepared by the prefectural governments by using large-scale trainings and human resource development. For example, in Aomori Prefecture, which is north of Fukushima, a large-scale drill was conducted in 2017 to verify whether contamination tests could be smoothly performed with the residents' participation (13), and workshops on the correct uses of the radiation measuring instruments have commenced throughout the country (30, 31).

This paper describes the nuclear disaster response system that evolved in Japan after the FDNPP accident and reported on preparedness at the prefecture level. Continuity is important to effective training and human resource development, and we argue that it is important for the many stakeholders involved in nuclear disaster prevention to know about each other's efforts and support each other through education. In addition, it is necessary for stakeholders to understand the importance of cooperation between the government and local governments and changes in initiatives depending on the disaster phase; this will help them guide residents. This paper aims to provide stakeholders with current information on nuclear disaster preparation in Japan, and it supports a future in which a wide variety of specialists work together to develop effective human resources and verify disaster response systems.

## Data Availability Statement

All datasets generated for this study are included in the article/supplementary material.

## Ethics Statement

The studies involving human participants were reviewed and approved by The ethics committee of the Graduate School of Health Sciences, Hirosaki University. Written informed consent for participation was not required for this study in accordance with the national legislation and the institutional requirements.

## Author Contributions

TT oversaw the summary, questionnaire, and writing of the manuscript. MS and TK formulated, distributed, and collected questionnaires and data, and YS, KO, KI, KY, and IK gave advice on the writing of the Japanese nuclear disaster response system and on questionnaire development. All authors contributed to the article and approved the submitted version.

## Conflict of Interest

The authors declare that the research was conducted in the absence of any commercial or financial relationships that could be construed as a potential conflict of interest.
